# Deterministic optical polarisation in nitride quantum dots at thermoelectrically cooled temperatures

**DOI:** 10.1038/s41598-017-12233-6

**Published:** 2017-09-21

**Authors:** Tong Wang, Tim J. Puchtler, Saroj K. Patra, Tongtong Zhu, John C. Jarman, Rachel A. Oliver, Stefan Schulz, Robert A. Taylor

**Affiliations:** 1 Department of Physics, University of Oxford, Parks Road, Oxford, OX1 3PU UK; 2 Tyndall National Institute, University College Cork, Cork, Ireland; 3 Department of Electrical Engineering, University College Cork, Cork, Ireland; 4 Department of Materials Science and Metallurgy, University of Cambridge, 27 Charles Babbage Road, Cambridge, CB3 0FS UK

## Abstract

We report the successful realisation of intrinsic optical polarisation control by growth, in solid-state quantum dots in the thermoelectrically cooled temperature regime (≥200 K), using a non-polar InGaN system. With statistically significant experimental data from cryogenic to high temperatures, we show that the average polarisation degree of such a system remains constant at around 0.90, below 100 K, and decreases very slowly at higher temperatures until reaching 0.77 at 200 K, with an unchanged polarisation axis determined by the material crystallography. A combination of Fermi-Dirac statistics and **k·p** theory with consideration of quantum dot anisotropy allows us to elucidate the origin of the robust, almost temperature-insensitive polarisation properties of this system from a fundamental perspective, producing results in very good agreement with the experimental findings. This work demonstrates that optical polarisation control can be achieved in solid-state quantum dots at thermoelectrically cooled temperatures, thereby opening the possibility of polarisation-based quantum dot applications in on-chip conditions.

## Introduction

The optical polarisation control of a quantum dot (QD) system is an essential requirement for a number of optoelectronic and quantum information applications, such as QD liquid crystal displays^1^, optical quantum computing^2^, and quantum key distribution^3–5^. These 0D nanostructures possess a delta function-like density of states similar to atoms, whilst being thousands of times larger. Since their size and shape can be manipulated in the solid-state, QDs offer an easier path towards integration and development on semiconductor platforms than other comparable systems, such as atoms^6^, molecules^7^, carbon nanotubes^8^, and material defects embedded in nitride wafer substrates^9^, diamond^10^, silicon carbide^11^, and 2D nanostructures^12,13^. However, in most QD systems, polarised light output can only be achieved using an external polarisation filter to define a desired polarisation state. Such configurations can be cumbersome and will incur a loss of at least 50%. To mitigate these undesired losses, direct polarisation control from the light source would be desirable. More importantly, for realistic scalable on-chip applications, one challenge that a QD system will inevitably face is the operation at temperatures high enough to reach the regime of on-chip electronic temperature regulation by thermoelectric cooling. Commercial Peltier coolers can maintain a stable temperature difference of ~100 K, allowing devices to work at ~190 K. As such, it is important not only to demonstrate QD properties, such as polarisation control, under cryogenic conditions, but also to investigate their performance and behaviour at thermoelectrically cooled temperatures. In this respect, nitride materials possess large band offsets and exciton binding energies^14^, and are suitable for operation at such high temperatures.

The polarisation of light emitted by a QD is governed by the underlying crystal structure and the QD geometry. In general, the dipole moment is preferentially enhanced parallel to the direction of anisotropy of the QD geometry in a simplified picture. This effect is also found to be stronger in nitrides than in systems based on other materials, such as CdSe or InAs^15–17^, owing to particularities of the III-N valence band structure^18,19^. As such, there have been several reports of polarised QD emission in conventional *c*-plane nitrides^20–26^ at cryogenic temperatures. However, due to the stochastic process of QD formation, the resultant extent and direction of anisotropy are completely random, giving rise to uncertain degrees of optical linear polarisation (DOLP) along arbitrary directions that are less than desirable in polarisation-based applications. A few attempts have been made to control the geometric strain of the QDs, including dots in nanowires^27–29^, elliptical nanowires^18^, and asymmetric pyramidal QDs^30^. Although the direction has been defined, the DOLP in nanowire-based systems is not particularly high due to the limited degree of anisotropy control (for elliptical nanowires) and nanowire-induced electric field attenuation orthogonal to its long axis (when a nanowire is aligned horizontally). For all these systems, the development of electrically pumped devices and higher temperature operation is challenging due to the need for direct contacting to the nanostructures and proper heat dissipation in restricted geometries.

In this work, we demonstrate both theoretically and experimentally a different approach to achieve linearly polarised emission at 200 K with a deterministic polarisation axis, by the use of non-polar *a*-plane InGaN QDs^31^. The use of a growth plane orthogonal to the wurtzite *c*-axis brings about system-wide symmetry breaking and connected valence band splitting effects, thereby producing linearly polarised emission along the crystal *m*-axis^32,33^. This effect overrides the randomness of anisotropy-induced polarisation properties and supersedes the need for nanostructure strain engineering in the realisation of polarisation control. With this planar system fabricated via metal-organic vapour phase epitaxy (MOVPE, see Methods), the challenges for heat dissipation and future electrical contacting have also been minimised. To understand the origin of these high temperature polarisation properties^34,35^ from a fundamental perspective, we combined Fermi-Dirac statistics and rigorous **k**·**p** modelling^33,36^ to investigate the polarisation properties of both the ground and higher excited states, and gained insights into the DOLP of *a*-plane InGaN QDs at elevated temperatures. We then couple these results with statistically significant experimental data to show that *a*-plane InGaN QDs are efficient polarised light emitters at temperatures in thermoelectrically regulated conditions.

## Results and Discussion

### Polarised emission at elevated temperatures

**Figure 1:**
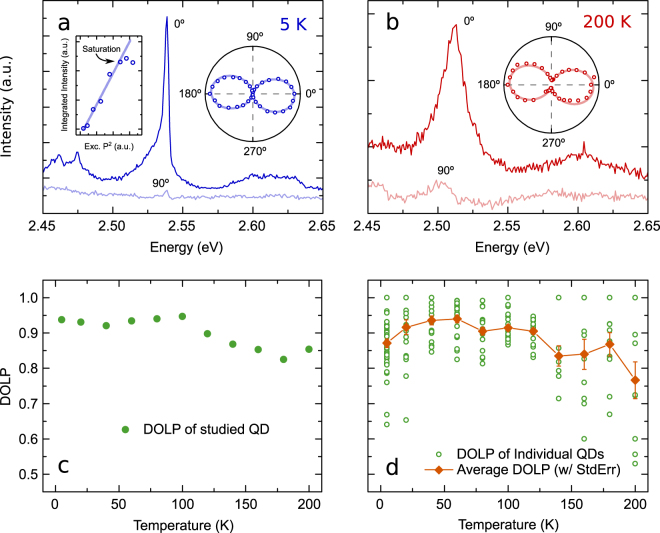
Experimental temperature-dependent polarisation-resolved μ-PL measurements of the DOLP of *a*-plane InGaN QDs. (**a**) PL spectra at maximum and minimum intensities of a single representative QD, which correspond to emission parallel and perpendicular to the crystal *m*-axis. Left inset: Linear power-squared dependence of PL intensity with laser excitation power. Right inset: QD emission intensities at each 10° polariser angle over a full 360° cycle. (**b**) Cross-polarised PL spectra with inset of polarisation-dependent intensity variation of the same QD measured at 200 K. **(c)** DOLP of the studied QD at 5 K, and from 20 to 200 K at 20 K intervals. With a slight reduction at higher temperatures, the DOLP always remained at 0.80 and above. **(d)** Statistical study of the DOLP variation (open circles) with temperature. Also shown is the average DOLP (filled diamonds) with standard error at each temperature step, which stays above 0.75.

We use microphotoluminescence (μ-PL, see Methods) to investigate the optical properties of the QDs. The polarisation-resolved μ-PL spectra of an *a*-plane InGaN QD emitting at a representative energy of ~2.54 meV at 5 K are shown in Fig. 1(a), since typical emission energies for our QDs range between 2.3 to 2.8 eV. The non-zero background signal across the spectral range in Fig. 1(a) is caused by the growth of an InGaN quantum well (QW) epilayer as part of the fabrication process, upon which QDs are formed. The intensity of the QD emission is found to increase linearly with the square of the excitation power before saturation takes place, as shown in the left inset of Fig. 1(a). Since we use two-photon excitation, this quadratic power dependence indicates that the emission originates from an exciton, rather than a biexciton with a fourth-power correlation. Biexcitonic emission from this particular dot was not observed, however such emission has been measured in other *a*-plane dots^37^. The emission intensity reaches a maximum when the polariser angle *θ* is set to 0°, which in our optical setup corresponds to a polarisation axis along the crystal [1–100] *m*-axis (perpendicular to the *c*-axis). The intensity of the QD gradually decreases as the polariser is rotated towards 90°, where a minimum is reached. The detailed intensities at 10° intervals over a full 360° cycle are shown in the right inset of Fig. 1(a) and fitted with the equation,

1 $$I(\theta )={I}_{\perp }{\cos }^{2}(\theta -\varphi )+{I}_{\parallel }{\sin }^{2}(\theta -\varphi ),$$

where ${I}_{\perp }$, ${I}_{\parallel }$ are intensities perpendicular and parallel to the crystal [0001] *c*-axis. Since both cross-polarised components should exhibit optical linear polarisation, it is important to also describe ${I}_{\parallel }$ with a sinusoidal function 90° out of phase with that of ${I}_{\perp }$. An analysis with $I(\theta )={I}_{\perp }{\cos }^{2}(\theta -\varphi )+{I}_{\parallel }$ alone would introduce errors in the DOLP calculation, especially for systems with non-unity DOLP. Equation (1) is thus equivalent to the Malus’ law of $I(\theta )=({I}_{\perp }-{I}_{\parallel }){\cos }^{2}(\theta -\varphi )+{I}_{\parallel }$, a sinusoidal function with a maximum of ${I}_{\perp }$ and a minimum of ${I}_{\parallel }$. Here, *ϕ* is an offset angle found to be ~ −10°. Striations along the *c*-axis, and thus knowledge of the orthogonal deterministic polarisation axis, can be distinctly observed by the naked eye without the use of any microscopy, a feature useful for characterisation and development. The −10° offset angle is attributed to the difficulty in perfectly aligning the sample with its *c*-axis perpendicular to the polariser’s fast axis. The peak intensities of the cross-polarised PL, along with the experimental DOLP formula^38^,

2 $$\rho =\frac{{I}_{\perp }-{I}_{\parallel }}{{I}_{\perp }+{I}_{\parallel }},$$

yield a polarisation degree of 0.93 at 5 K, which is close to the average DOLP we observe in *a*-plane QDs^33^. At 200 K, the emission of the studied QD redshifts to ~2.51 meV. The linewidth broadening of the excitonic transition observed in Fig. 1(b) can be quantified as an increase from 3.39 ± 0.87 to 20.02 ± 1.23 meV, caused by both higher degrees of acoustic phonon coupling and a greater degree of spectral diffusion^29,34^. The sinusoidal intensity variation and fitting with Eq. (1) shown in the inset of Fig. 1(b) again indicates the emission of linearly polarised photons, with an unchanged polarisation axis. Calculations with Eq. (2) yields a slightly reduced *ρ* of 0.85, demonstrating highly polarised emission with a deterministic direction above the thermoelectric cooling barrier.

As the temperature was increased, the maximum and minimum intensities of the studied QD were recorded at 20 K steps up to 200 K, and the DOLP calculated is shown in Fig. 1(c). At each step, the temperature was allowed to stabilise to an error of <1 K of the desired value, before measurements were taken. The polariser was rotated through a 360° cycle and the intensity variation was observed. The angles at which the highest and lowest intensities occur at each step remain the same, indicating no polarisation direction changes during the temperature increase. In Fig. 1(c), the polarisation degree stays fairly constant up to 100 K. The DOLP then starts to decrease slowly at higher temperatures, reaching 0.85 at 200 K. This small decrease (0.08), and consequent insensitivity to changing temperature, indicate that the studied *a*-plane InGaN QD is indeed a reliable polarised photon emitter even at thermoelectrically cooled temperatures. We will address the origin of this decrease later in the theory section.

In order to achieve statistical significance for this finding, we proceed to investigate the polarisation properties of ~200 individual QDs with no selection bias at different temperatures. In particular, 40 QDs at 5 K, 20 QDs at 20 K intervals from 20 to 120 K, and 10 QDs at 20 K intervals from 140 to 200 K were studied. Overlapping of data points exists in Fig. 1(d) due to identical values, especially at higher temperatures where the integer count rates are much lower (see Data access for details). For each step, QDs were randomly selected in order to assess the global polarisation characteristics of *a*-plane QDs at that temperature. An average DOLP (filled diamonds) with standard error at each temperature was computed and is displayed with the DOLP data for individual QDs (open circles) in Fig. 1(d). The average stays above 0.85 and remains relatively constant from 5 to 120 K, before small drops occur beyond this temperature range. In fact, around half of the measured QDs have a DOLP of 0.9 or higher for T ≤ 100 K. It is worth noting that from 5 to 200 K, we have measured several QDs with a DOLP of 1, and a few more very close to 1 at each temperature step. These QDs could hence be selected for applications demanding 100% polarisation efficiency. Conversely, we also find QDs with lower DOLP values. We will come back to the effects that might have caused these very different behaviours when we study the fundamental properties of non-polar InGaN QDs from a theoretical perspective. Nonetheless, all average DOLP data remain at or above 0.77, indicating statistically high DOLP up to 200 K, comparable even to most low-temperature QD DOLP reports from the literature^15–18,20,21,23,27–29^. The direction of polarisation of these QDs also coincides with the crystal *m*-axis (perpendicular to the *c*-axis) at all temperatures studied here.

The spread of the DOLP values at each temperature step could be attributed to the effect of QD geometry. It is worth noting that the spreads of the distributions at T ≤ 120 K in Fig. 1(d) are reasonably narrow. With the exception of 3 QDs with a DOLP of ~ 0.65, all other (~150) single QDs exhibit a DOLP between 0.75 and 1. At T > 120 K, the DOLP values become more widespread (0.5 to 1), and the number of emitting structures available to study dropped. Due to quantum confinement effects, one can expect that the number of emitting QDs reduces since only those with strong confinement should still be radiatively active at elevated temperatures. The larger spreads observed at higher temperatures are indicative of the presence of lower DOLP for some QDs in this temperature range. As such, our data indicate that features of both ground and excited states would play a central role in explaining the observed variations in the DOLP. Thus, a detailed understanding of the electronic structure of non-polar *a*-plane QDs is key. Furthermore, the spread of these values suggests that QDs with different structures, for example anisotropies, might result in a greater variation of polarisation characteristics at high temperatures. Hence, rigorous theoretical investigation of the relationship between QD anisotropy, DOLP, and temperature is required to elucidate the origin of this behaviour.

### Theoretical study on the impact of temperature and QD geometry on DOLP

Having discussed the temperature dependence of DOLP from an experimental point of view, we now address the question from a theoretical perspective. In doing so, we also gain insight into the underlying physics plus the theoretical framework that can be used to guide future designs of non-polar InGaN/GaN QDs with further improved temperature stability of the DOLP. To shed light onto these questions, we analysed, using multi-band **k**·**p** theory^39,40^, the spontaneous emission rates of non-polar InGaN/GaN QDs. The spontaneous emission rate *R*_sp_ is given by^41^:

3 $${R}_{sp}=\int d(\hslash \omega )\frac{2{e}^{2}n\hslash \omega }{{m}_{0}^{2}{\varepsilon }_{0}{c}^{3}{\hslash }^{2}}{\sum _{i,j}|{\bf{a}}\cdot {{\bf{p}}}_{i,j}|}^{2}\frac{1}{\sqrt{2\pi }\sigma }\exp [\frac{-{({\rm{\Delta }}{E}_{i,j}-\hslash \omega )}^{2}}{2{\sigma }^{2}}]\,{f}^{e}({E}_{i}^{e}){f}^{h}({E}_{j}^{h}).$$

Here, *e*, ${\varepsilon }_{0}$, *m*_0_, *c*, $\hslash$, *n* denote elementary charge, vacuum permittivity, free electron mass, vacuum speed of light, reduced Planck’s constant, and the refractive index, respectively. The energetic separation between the electron state *i* and the hole state *j* is given by ${\rm{\Delta }}{E}_{i,j}$ (transition energies). The inhomogeneous broadening parameter is denoted by *σ*. Experimentally, the values of full width at half maximum (linewidth) for the *a*-plane InGaN/GaN QDs studied here are on the order of 0.5–3 meV at low temperatures^42^. Given these extremely small numbers compared to *a*-plane InGaN/GaN QWs^32^, inhomogeneous broadening is neglected. The momentum matrix element between electron state *i* and hole state *j* is given by ${|{\bf{a}}\cdot {{\bf{p}}}_{i,j}|}^{2}$, which also contains the light polarisation vector **a**. The Fermi-functions for electrons and holes are denoted by *f* ^*e*^ and *f* ^*h*^ respectively (see Methods). These functions in Eq. (3) account for the effect that with increasing temperature *T*, excited electron and hole states are populated. Given that *R*_sp_ depends on **a** and *T* via the Fermi-functions, we can now define the theoretical temperature dependence of the DOLP *ρ* by

4 $$\rho (T)=\frac{{R}_{sp}^{\perp }(T)-{R}_{sp}^{\parallel }(T)}{{R}_{sp}^{\perp }(T)+{R}_{sp}^{\parallel }(T)}.$$

The spontaneous emission rate for **a** perpendicular and parallel to the wurtzite *c*-axis are denoted by ${R}_{sp}^{\perp }$ and ${R}_{sp}^{\parallel }$ respectively. This approach is similar to that used in previous work^43^ for non-*c*-plane InGaN/GaN QWs. More details about our theoretical framework are given in Methods.

**Figure 2:**
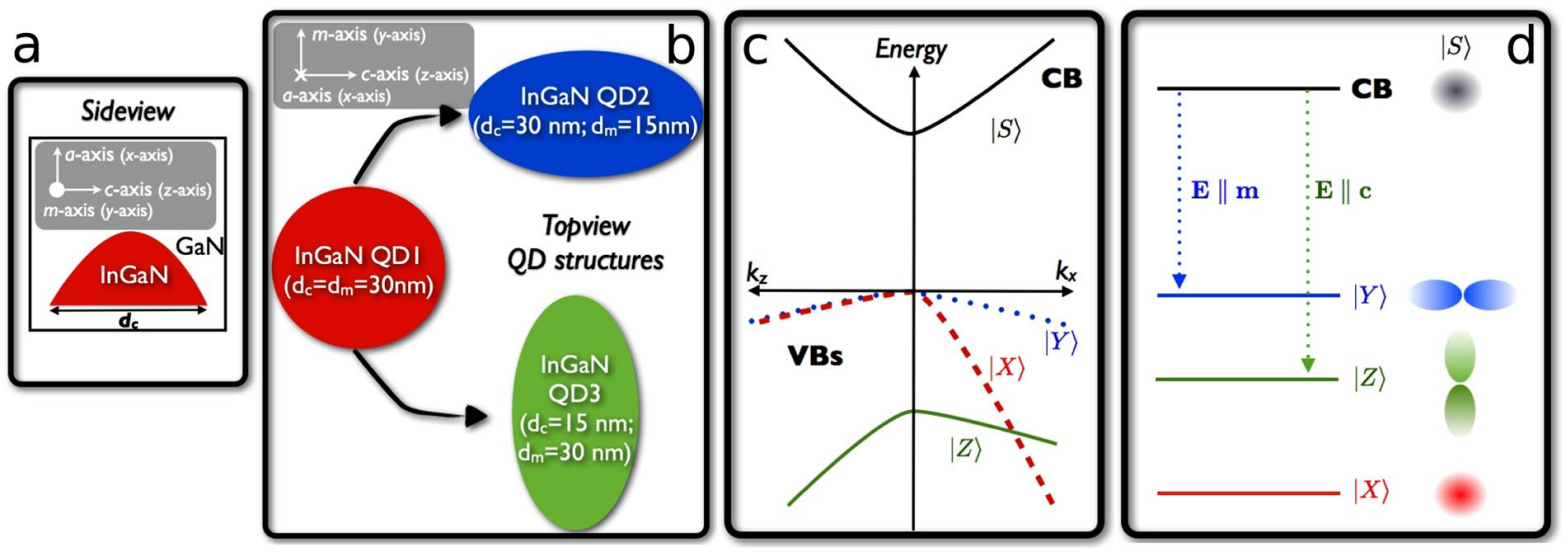
Schematic illustrations of the *a*-plane InGaN/GaN nanostructures investigated and relevant energy diagrams in discussion. (**a**) Side view (perpendicular to the *c*- and *a*-axis) of the lens-shaped QD assumed in this work. (**b**) Top view (parallel to the *a*-axis) of the three different QD geometries (QD1, QD2, QD3) studied here. (**c**) *c*-Plane bulk band structure in the absence of spin-orbit coupling. (**d**) Energy level ordering of an *a*-plane system with confinement along the *a*-axis (*x*-axis). Selection rules for the electric field **E** (light polarisation vector **a**) parallel to the *c*- (*z*−) and *m*-axis (*y*-axis) are also indicated by the dashed arrows. A schematic illustration of the involved orbitals is on the right-hand side.

To calculate the DOLP *ρ*(*T*) as defined by Eq. (4), information about the QD geometry is required. In the following section, we have assumed a lens-shaped dot with a base diameter of 30 nm and a height of 2.5 nm as the model geometry, with indium content set to 20%. *ρ*(*T*) is a quantity strongly dependent on the wavefunction character of the hole states and thus band mixing effects. Since we are interested in how different anisotropies could have caused the spread of experimental DOLPs at high temperatures, the in-plane geometry of the non-polar In_0.2_Ga_0.8_N/GaN QDs has been varied. More specifically, the height of the QD is fixed at *h* = 2.5 nm, while the in-plane dimensions have been varied between 30 nm and 15 nm. Hence, three different QD structures, labelled as QD1, QD2, and QD3, have been analysed. QD1 has a circularly symmetric base where the dimensions *d*_*c*_ and *d*_*m*_ along the *c*-axis (*z*-axis) and *m*-axis (*y*-axis) respectively are both 30 nm (*d*_*c*_ = *d*_*m*_ = 30 nm). A schematic illustration of the QD geometry is given in Fig. 2(a). Since such a symmetric system is unlikely for realistic structures, the dimensions along the *m*- and *c*-axis have been halved for QD2 and QD3 respectively (QD2: *d*_*c*_ = 30 nm, *d*_*m*_ = 15 nm; QD3: *d*_*c*_ = 15 nm, *d*_*m*_ = 30 nm), as illustrated in Fig. 2(b). Due to the underlying crystal structure, similar QD elongations have been observed in other non-polar nitride-based QD systems^44^. More details about the choice of QD dimension and geometry are given in Methods. Overall, using these settings, we obtained ground state transition energies in the range of 2.7 to 2.9 eV, which are in reasonable agreement with our experimental findings explained above, and available literature data^31,33–35,37,42,45,46^.

Before turning to the calculated *ρ*(*Τ*) values, it is important to discuss and understand general band structure features of non-polar *a*-plane wurtzite InGaN/GaN heterostructures, given that *ρ*(*Τ*) depends on the orbital character of the involved electron and hole states *i* and *j* (cf. Eqs (3) and (4)). In a first step, we start with a general discussion of the *c*-plane bulk system, where |*X*〉- and |*Y*〉-like states are energetically degenerate^47^ at **k** = 0, neglecting the weak spin-orbit coupling. Due to the positive crystal field splitting energy in InN and GaN^48^, the |*Z*〉-like state is shifted to lower energies. To understand how quantum confinement affects the band structure of InGaN/GaN heterostructures, the effective masses of |*X*〉-, |*Y*〉-, and |*Z*〉-like states near **k** = 0 are of central importance. Whilst the energy bands associated with |*X*〉- and |*Z*〉-like states have low effective masses along *k*_*x*_- and *k*_*z*_-directions respectively, the |*Y*〉-like state has a light-hole mass along the *k*_*y*_-direction^48,49^. A schematic illustration of the situation is given in Fig. 2(c). Thus, for a system with a strong confinement along the *c*-axis (*z*-axis), such as *c*-plane InGaN/GaN QWs, |*Z*〉-like states are shifted to lower energies with respective to the valence band maximum. Therefore, the topmost valence band state in such a system is |*X*〉- and |*Y*〉-like with no |*Z*〉-orbital character. In the case of *c*-plane QDs, additional confinement effects are introduced by the lateral/in-plane (*x*-*y*-plane) confinement. Given the difference in the effective masses of |*X*〉- and |*Y*〉-like states, QD shape anisotropies will significantly affect band mixing effects and thus the orbital character of the QD hole states^50^, especially for excited states. Consequently, the DOLP and its temperature dependence will be strongly affected.

Turning now to the non-polar *a*-plane system, the situation becomes different. Since growth is along the *a*-axis, perpendicular to the *c*-axis, the symmetry between |*X*〉- and |*Y*〉-like states is broken given the strong confinement along the *a*-axis (*x*-axis). This results in the situation that the |*X*〉-like state is shifted to lower energies with respect to the |*Y*〉-like state. Thus, the topmost valence band state is predominantly |*Y*〉-like in character taking into account that the |*Z*〉-like state is already shifted to lower energies due to the positive crystal field splitting energy. A schematic illustration of the general valence band state ordering in a system with confinement along the *a*-axis (*x*-axis) is given in Fig. 2(d). Please note that in the following we apply the *c*-plane convention for the coordinate system, meaning that the growth direction is assumed to be along the *x*-axis. The in-plane axes are the *z*- and *y*-axes. Overall, using the *c*-plane convention discussed above and assuming growth along the *x*-axis, we expect that the hole ground state in an *a*-plane InGaN/GaN QD with a circular symmetric base is predominantly |*Y*〉-like in character. However, depending on the in-plane QD shape anisotropies, band mixing effects are expected to play an important role for excited states, which are key for the DOLP at elevated temperatures (cf. Eq. (3)).

**Figure 3:**
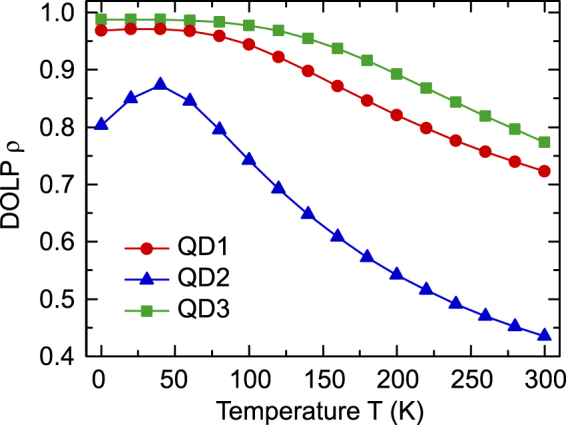
Theoretically calculated DOLP *ρ* as a function of the temperature T. QD1, QD2, and QD3 correspond to three geometries of lens-shaped *a*-plane In_0.2_Ga_0.8_N/GaN QDs, as detailed in Fig. 2(a) and (b). An elongation along the *c*-axis (QD2) decreases the DOLP temperature stability while that along the *m*-axis (QD3) enhances it. QD1 and QD3 have very insensitive temperature dependence of DOLP up to 100 K. However, it should be noted that even for T > 100 K, all calculated values are still very high (>0.70).

Equipped with a knowledge of the basic features of the band structure, we now turn to analyse the temperature dependence of the DOLP *ρ*. Figure 3 displays the calculated *ρ* values as a function of *T* for the three different QD geometries discussed above. We start our analysis with the symmetric QD, i.e. QD1 (cf. Figure 2(a)). The corresponding data for *ρ* are given by the red circles in Fig. 3. For low temperatures ($T\le 20$ K) the DOLP value is extremely high ($\rho (20)\approx 0.96$). Following our discussion above, the hole ground state is expected to be predominantly |*Y*〉-like in character, which is confirmed by our calculations. Furthermore, given the low temperature of *T* = 20 K, contributions from excited states are negligible, resulting in a high DOLP value of 0.96. We observed here that *ρ* stays approximately constant up to 80 K. Over this temperature range mainly states with a large percentage of |*Y*〉-like orbital character are populated, and thus ${R}_{sp}^{\perp }\gg {R}_{sp}^{\parallel }$. Above 80 K, we observe that contributions from states with a higher percentage of |*Z*〉-like character become important. This results in the situation that ${R}_{sp}^{\perp }$ decreases with increasing *T*, while ${R}_{sp}^{||}$ starts to increase. Consequently, based on Eq. (4), *ρ* starts to decrease with increasing temperature. In the case of QD1, at thermoelectrically cooled temperature (200 K), *ρ* is reduced to approximately 0.82. At room temperature (300 K), this value drops to 0.71.

As already mentioned above, such a symmetric dot is unlikely given the asymmetry of the *a*-plane in terms of the underlying *c*- and *a*-lattice constants^48^, and the stochastic self-assembly formation process. Thus, we now focus our attention on QD2 and QD3, where an anisotropy along the *c*- and *m*-directions are present. For QD2, the in-plane dimension along the *m*-axis (*y*-axis), perpendicular to *c*-axis, is reduced to 15 nm (*d*_*m*_ = 15 nm), while keeping the dimension along the *c*-axis (*z*-axis) constant at *d*_*c*_ = 30 nm (cf. Fig. 2(b)). Based on our discussion of the valence band structure and the involved effective masses, the band mixing effects between |*Y*〉- and |*Z*〉-like states in this situation are expected to increase. This originates from the fact that states with a larger |*Y*〉-like orbital contribution should be much more strongly affected by the increased confinement effect along the *m*-axis (*y*-axis) when compared with predominately |*Z*〉-like states. Consequently, in comparison to the symmetric QD (QD1), one could expect that at low temperatures *ρ* is reduced, accompanied by an earlier onset of the reduction in *ρ* with increasing temperature. This behaviour is indeed reflected in the calculated values for *ρ*(*T*) of QD2, which are given by the blue triangles in Fig. 3. In comparison to QD1, we find lower DOLP values at $T\le 20$ K. Additionally, *ρ* starts to decrease rapidly at $T\ge 40$ K. At 200 K, the *ρ* of QD2 drops to 0.54. This low value is consistent with those experimental DOLP data between 0.5 and 0.6 in Fig. 1(d), indicating that indeed strong shape anisotropies might be present for QDs with very low DOLP values.

Making use of the insight gained into the band structure and how the QD geometry affects the temperature dependence of the DOLP, we can now provide a potential way forward to improve the temperature stability of *ρ*(*T*) further. Given the positive crystal field splitting energy and taking into account the light-hole mass of predominantly |*Z*〉-like states along the *c*-axis (*z*-axis), greater confinement along the *c*-axis should decrease band mixing effects between |*Y*〉- and |*Z*〉-like states, which would be especially relevant for excited hole states. Consequently, by reducing the dimension of the QD along the *c*-axis, *ρ* should stay approximately constant over a wider temperature range when compared to QD1 and QD2. Also, the DOLP *ρ* should be slightly larger at low temperatures in comparison with the symmetric dot QD1, for instance. To verify this prediction, we have performed calculations, for an elongated QD which is labelled as QD3 (cf. Fig. 2(b)). In Fig. 3, the green squares show the corresponding *ρ* values as a function of *T*. As expected from our analysis above, *ρ*(*T*) for QD3 is always higher than in the case of QD1 (red circles) and QD2 (blue triangles). Furthermore, for QD3 up to 200 K, the drop in *ρ*(*T*) is strongly reduced in comparison to QD2 and still distinctly smaller with respect to QD1. Moreover, for QD3, *ρ*(*T*) stays approximately constant ($\rho \approx 0.98$) up to 100 K, before it starts to slightly decrease over the temperature range of 100 to 200 K. At 200 K, we find a very high value of $\rho (200)\approx 0.90$. Even up to room temperature (300 K), the emission is still strongly polarised with $\rho (300)\approx 0.77$.

Our findings for QD1 and QD3 are in good agreement with the experimental *ρ* data on the single *a*-plane InGaN QD displayed in Fig. 1(c). The experimental study reveals that up to 100 K the average DOLP is approximately constantly around 0.95, and slightly decreased to a value between 0.80 and 0.85 at T > 100 K. The three QDs studied theoretically can also be used to explain the spread of the experimental values at higher temperatures in Fig. 1(d), where differences in anisotropy could have resulted in the DOLP range of 0.5 to 1.0 at 200 K. Although the control of QD anisotropy is not very straightforward, it is worth noting that control of nanostructure aspect ratio has been achieved in conventional III-V semiconductors, e.g. using miscut substrates to encourage the formation of quantum dashes^51^. Our theory thus provides a guideline to target asymmetric QD structures for achieving emitters with a further improved temperature stability of the DOLP *ρ*. For example, with QDs squeezed along the *c*-axis (elongation along *m*-axis), we would be able to achieve even higher polarisation degrees at ambient conditions.

## Conclusion

We have demonstrated the emission of linearly polarised light with an easily identified, deterministic polarisation axis from non-polar *a*-plane InGaN QDs up to the thermoelectric cooling regime of 200 K. Our combined theoretical studies in Fermi-Dirac statistics and **k** · **p** theory show that the polarisation degree of this system is relatively insensitive to both anisotropy and temperature changes, thanks to the reduced band mixing effect caused by the change in symmetry in *a*-plane structures. Statistically significant experimental investigations demonstrate highly polarised emission with an average DOLP of >0.9 at lower temperatures, and 0.77 at 200 K, which agrees very well with the theoretical results. The statistical low-to-medium temperature result is already higher than nanowire-based nitride polarisation control systems^27–29^, and marginally higher than the aforementioned nitride pyramidal structure that requires considerable strain engineering^30^. Comparing to polarised QD systems *without* a deterministic polarisation axis, the statistical average DOLP is one of the highest among reports where a single selection of QD result is shown^20–26^. To the best of our knowledge, *a*-plane InGaN is also the first solid-state QD system to directly demonstrate highly polarised emission with a deterministic axis at temperatures reachable by thermoelectric cooling. In fact, no reports on other QD systems have considered polarisation theoretically or demonstrated the successful generation of polarised light experimentally at any temperature above cryogenic conditions. Based on our theoretical and experimental findings, we also expect the future development of *a*-plane InGaN QDs elongated along the crystal *m*-axis to have > 0.9 DOLP at room temperature. The results presented in this work as a whole shows that efficient polarisation control in on-chip temperatures can be achieved in solid-state QDs, a step forward for the development or related optoelectronic and quantum information applications.

## Methods

### Sample fabrication and optical characterisation

The non-polar *a*-plane InGaN/GaN QDs were grown by MOVPE on *r*-plane sapphire substrate in a 6 × 2-inch Thomas Swan close-coupled showerhead reactor using the recently discovered quasi-two temperature method^45^. After the growth of an InGaN epilayer (~16 monolayers thick) at 690 °C, a ~2 nm thick GaN cap was initially grown at the InGaN growth temperature, the GaN capping process continued as the temperature was ramped in 90 s to 860 °C, at which the GaN capping growth was completed. The QD formation mechanism is similar to Stranski-Krastanov mode, where the resultant structures contain an InGaN QW layer with a number of QDs on it^45^. Such QD growth method can produce a much smoother underlying InGaN QW with fewer non-radiative escape pathways, and thus exhibit improved temperature stability compared to the alternative modified droplet epitaxy (MDE) method^31^. To isolate individual QDs and increase the photon extraction efficiencies, nanopillars with a diameter of ~180 nm were fabricated using drop-cast silica nanospheres as the etch mask and inductively coupled plasma etching^33^.

Temperature-dependent polarisation-resolved μ-PL experiments were performed on these samples to assess their temperature performance and polarisation properties. Visible striations along the sample surface were used to determine the crystal *c*-axis, and the sample was aligned such that emission parallel to its *m*-axis corresponded to 0° of the polariser in the optical collection path, within a maximum error of 10°. The cryostat is an AttoDRY800 close-cycle system, capable of maintaining the sample temperature within an error of <15 mK from 5 to 320 K. Attocube positioners were used to control the movement of the sample, as well as to maintain alignment between a QD and the normally incident laser beam during temperature changes. 800 nm, 1 ps pulses from an 80 MHz repetition-rate Ti:Sapphire laser was used to provide pulsed two-photon excitation, a technique to enhance the QD/QW ratio^52^ for greater accuracy of optical characterisation. The laser beam was focused by a 100 × NIR objective with 0.5 N.A., producing a ~1 μm spot on the sample. In the optical collection, a polariser and a half-wave plate were used for polarisation studies. Each angle *θ* rotated on the polariser is followed by a -*θ*/2 rotation on the half-wave plate, so that the direction of the resultant PL remained parallel to the slit of a half-metre Shamrock 500i spectrometer. The polarised PL was eventually dispersed by a 1200 l/mm grating, and detected by a thermoelectrically cooled Andor iDus 420 charge-coupled device with a background noise level of ~5 counts s^−1^.

### Theoretical framework

The temperature dependence of DOLP has been addressed in the present work on the basis of spontaneous emission rate *R*_sp_ in Eq. (3)^41^. As input for *R*_sp_, the energy levels for electrons and holes, ${E}_{i}^{e}$, ${E}_{j}^{h}$, and the momentum matrix element ${|{\bf{a}}\cdot {{\bf{p}}}_{i,j}|}^{2}$ from the electron and hole states *i* and *j* are required. Consequently, insights into the electronic structure of non-polar InGaN/GaN dots is of central importance. To this end, we have performed **k** · **p**-based calculations. For the investigation of the DOLP in non-polar *a*-plane InGaN/GaN QDs, a detailed understanding of the valence band structure, including related band mixing effects, is key. To capture these effects accurately, we employ a six-band Hamiltonian to describe the hole states. Electrons are treated by means of a single-band effective mass approximation. All calculations have been performed on a 50 × 50 × 30 nm^3^ supercell with periodic boundary conditions. The **k** · **p**-based model along with the underlying S/PHI/nX framework is described in detail elsewhere^36,40,53–56^.

Equipped with the knowledge about the electronic structure of the nanostructure, the momentum matrix elements ${|{\bf{a}}\cdot {{\bf{p}}}_{i,j}|}^{2}$ can be calculated via

5 $${|{\bf{a}}\cdot {{\bf{p}}}_{i,j}|}^{2}={\sum _{c,\upsilon }|\langle {u}_{c}|{\bf{a}}\cdot {{\bf{p}}}_{i,j}|{u}_{\upsilon }\rangle |}^{2}{|\langle {{\rm{\Phi }}}_{c}^{i}|{{\rm{\Phi }}}_{\upsilon }^{j}\rangle |}^{2},$$

where **a** is the polarisation of the incident light, *u* denotes the Bloch functions, and Φ is the envelope function. The subscripts *c* and *υ* denote conduction (electron) and valence (hole) states, respectively. Details on the calculation of these quantities have been addressed in the literature^57^.

To obtain the temperature dependence of *ρ* via Eq. (4), one needs to address the fact that with increasing temperature *T*, carriers start to occupy excited electron and hole states. In *R*_sp_, this is accounted for by Fermi-Dirac statistics via the Fermi-functions for electrons *f* ^*e*^ and holes *f* ^*h*^:

$$\begin{array}{c}{f}^{e}({E}_{i}^{e})=\frac{1}{1+\exp [({E}_{i}-{E}_{fn})/{k}_{B}T]},\\ {f}^{h}({E}_{j}^{h})=\frac{1}{1+\exp [({E}_{fp}-{E}_{j})/{k}_{B}T]}.\end{array}$$

Here *E*_*fp*_ and *E*_*fn*_ are quasi-Fermi levels obtained from the injected carrier density. The electron and hole energies for state *i* and *j* are denoted by *E*_*i*_ and *E*_*j*_ respectively. In accordance with the experiment, we deal only with excitonic effects, meaning that we assume one electron-hole pair per QD. To obtain the quasi-Fermi levels for electrons and holes up to 300 K, we include 10 electron and 30 hole states (neglecting the Kramers degeneracy of each state) in the calculations.

As discussed in the Results section, the theoretical framework requires the QD geometry and the indium content as input. Experimentally, it presents a nontrivial task to provide insights into these questions, especially after the QD structures are capped by GaN^58^. Atomic force microscopy investigations on uncapped *a*-plane InGaN QD structures reveal QD heights of the order of 7±3 nm^45^. For *capped polar* InGaN/GaN QDs, lens-shaped structures with heights of 3–4 nm and diameters between 25–40 nm have been reported in the literature^59^. This information presents the starting point for our calculations in terms of dot size and shape. It should be noted that previous calculations on *non-polar* InGaN/GaN QDs have made similar assumptions for the dot geometry^60,61^.

### Data Availability

The data presented in the Fig. 1 and 3 in this paper are available free of charge via the Internet at http://dx.doi.org/10.5287/bodleian:y0qp56MaD.
